# Combined hepatocellular-cholangiocarcinoma: a population level analysis of incidence and mortality trends

**DOI:** 10.1186/s12957-019-1586-8

**Published:** 2019-02-27

**Authors:** Jiakun Wang, Enliang Li, Hao Yang, Junjun Wu, Hong cheng Lu, Chenhao Yi, Jun Lei, Wenjun Liao, Linquan Wu

**Affiliations:** 1 Department of General Surgery, Second Affiliated Hospital of Nanchang University, Nanchang, China; 2 Department of Hepatobiliary and Pancreatic surgery, The Second Affiliated Hospital, Zhejiang University School of Medicine, Hangzhou, China; 3 Department of Breast Surgery, Second Affiliated Hospital of Nanchang University, Nanchang, China; 4 Department of Hepatobiliary Surgery, The Second Affiliated Hospital of Nanchang University, Nanchang, China

**Keywords:** Combined hepatocellular-cholangiocarcinoma (cHCC-CC), Annual percent change (APC), Trend, Survival analysis

## Abstract

**Background:**

The purpose of this study was to explore trends in incidence, incidence-based (IB) mortality, and survival for combined hepatocellular-cholangiocarcinoma (cHCC-CC) utilizing a population-based database to attract people’s attention to this disease.

**Methods:**

The Surveillance, Epidemiology, and End Results (SEER) database was utilized to investigate the incidence and IB mortality for cHCC-CC from 2000 to 2014. Trends in age-adjusted incidence and IB mortality were characterized by the Joinpoint Regression program. The Kaplan-Meier method and log-rank test were utilized to implement survival analyses. Cox regression was utilized to estimate independent predictors of mortality.

**Results:**

The incidence of cHCC-CC was 0.26 per 1,000,000 individuals in 2000 and 0.59 per 1,000,000 individuals in 2014, with an annual percent change (APC) (i.e., the extent of increase in incidence) of 3.84% (95% confidence interval [CI] 1.7–6.1; *P* < 0.05). The IB mortality also displayed a sustained increase (APC was 4.59%, 95% CI 1.9–7.4; *P* < 0.05). Compared to patients not undergoing surgery, patients undergoing surgical treatment experienced a significant increase in median survival (3 vs. 28 months; *P* < 0.001). However, the median survival decreased in patients with tumor size > 5 cm (20 vs. 9 months; *P* < 0.001). Based on univariate Cox regression analysis, African-American race, distant stage, regionalized stage, tumor size ≥ 5 cm, and no surgery were risk factors for death.

**Conclusions:**

We identified an overall steady increase in the incidence of cHCC-CC, which indicates that primary prevention strategies for cHCC-CC have not improved much in recent years and that cHCC-CC needs to be taken seriously.

## Background

Combined hepatocellular-cholangiocarcinoma (cHCC-CC) is a distinct type of primary hepatic cancer distributed across all races in the world. cHCC-CC was first reported by Allen in 1949 [1] and comprises ingredients of hepatocellular carcinoma (HCC) and intrahepatic cholangiocarcinoma (ICC) [2]. The histological features of cHCC-CC include hepatocytes that produce bile and bile duct epithelial cells that produce mucin [3].

At present, the gold standard of cHCC-CC diagnosis is histopathological examination, and the histopathological findings in biopsy specimen are that two progenitor cells of monoclonal origin are differentiated into hepatocytes and bile duct cells, which are closely integrated in the same tumor cell [4–6]. However, preoperative diagnosis of cHCC-CC is hard because of its particular imaging characteristics with similar features of HCC and ICC [7]. Although there may be a few features implying cHCC-CC on enhanced computer tomography and enhanced magnetic resonance imaging, previous studies have indicated that cHCC-CC are often misdiagnosed as either HCC or ICC [8, 9]. In addition, Portolani N showed that accuracy of cHCC-CC diagnosis in preoperative percutaneous liver biopsy is 11.1%, and the remaining cases are misdiagnosed as metastatic carcinoma, HCC, or ICC [10]. Currently, there is no unified standard for the diagnosis and treatment of cHCC-CC. Treatment of cHCC-CC mainly includes hepatic resection, liver transplantation (LT), transarterial chemoembolization (TACE), radiofrequency ablation (RAF), and percutaneous ethanol injection.

Unlike those of HCC or ICC, there is limited understanding of the clinical and prognostic features of cHCC-CC. Previous publications have been largely based on case series and retrospective studies which involve small research object populations from single institutions with finite statistical power [11–20]; thus, there is a lack of effective clinical evidence to help standardize the diagnosis and treatment and improve the prognosis of patients with cHCC-CC. In addition, most of these studies are histopathological in nature, and the demographic and prognostic features of the disease remain unclear. Despite improvements in and utilization of imaging technologies, operation method, combined adjuvant therapy, the incidence, and survival rates of cHCC-CC remain unknown. Therefore, the Surveillance, Epidemiology, and End Results (SEER)-18 database (2000–2014) was used to explore trends in the incidence and incidence-based (IB) mortality and survival for cHCC-CC. We also examined the independent predictors of mortality.

## Methods

### Data source

All data of patients with cHCC-CC between 2000 and 2014 were accessed from the SEER database from the National Cancer Institute (NCI). We used the SEER-18 database, which represents nearly 28% of the US population [21], to collect data on incidence, IB mortality and detailed patient characteristics (patient demographics, tumor and disease characteristics, survival of individuals with cancer).

### Study population

**Fig. 1:**
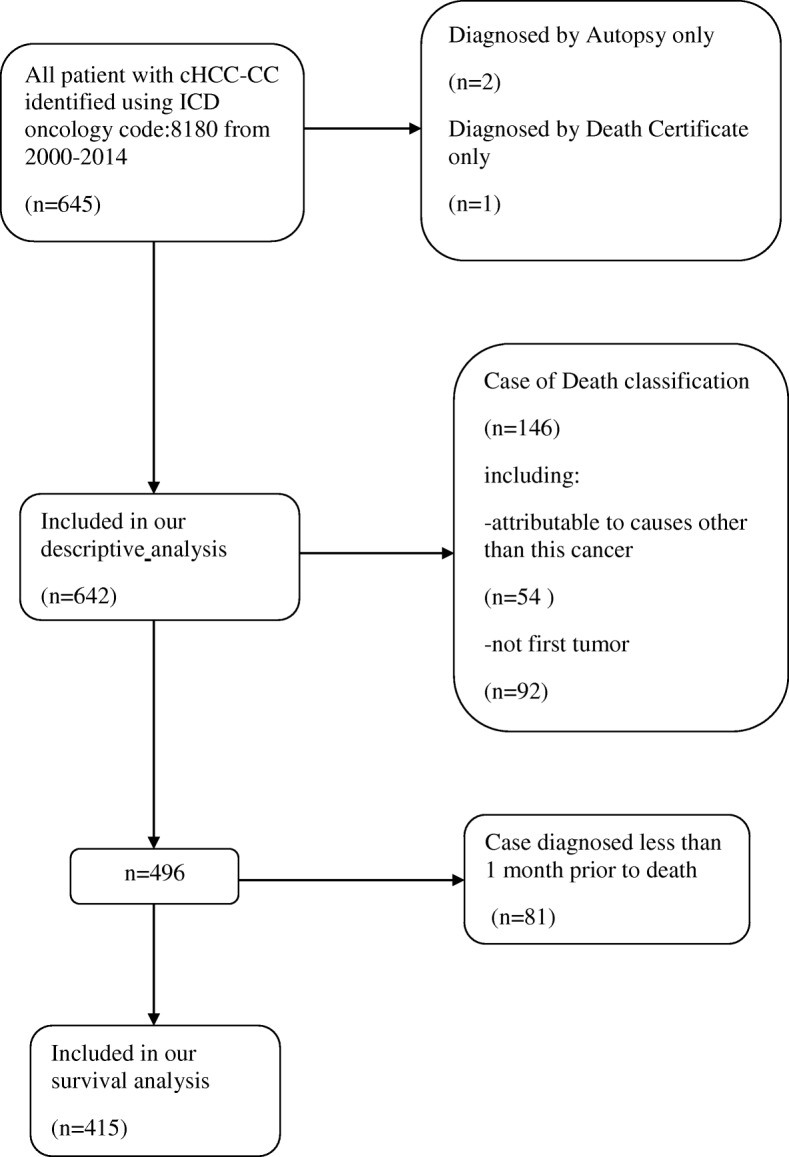
Flow diagram of patient selection out of the total 645 patients in the SEER database 2000–2014

We used the International Classification of Disease (ICD) for Oncology codes (8180) to select patients with cHCC-CC from 2000 to 2014 from the SEER database. These patients who died within 1 month of diagnosis were included in the descriptive analysis and trend analysis but precluded from the survival analysis, because the minimum unit of survival time was 1 month in the SEER database. If we used these cases, we would have survival data representing 0 month, which are not useful for our analyses. In addition, we excluded patients who died from other tumors from the survival analyses, which could have biased the effects of disease on survival (Fig. 1). As for the SEER stage classification, a tumor is depicted as a local stage which is confined to primary site, regional stage which is spreading to regional lymph nodes, distant stage which is already moving to the distance.

### Statistical analyses

We used the SEER*Stat software (version 8.34) to acquire data on incidence, IB mortality, and survival. We did not calculate standard mortality because the death certificate does not include the histological information of the tumor. Therefore, we combined information on the incidence of cHCC-CC with that on the death certificate to obtain data on IB mortality [22, 23]. In a prescribed year, the IB mortality for cHCC-CC is a part of the total deaths caused by cHCC-CC. The individuals considered to have died of cHCC-CC must have been diagnosed with cHCC-CC before death rather than being diagnosed at autopsy. Incidence and IB mortality are age-adjusted to the standard population in the USA in 2000.The Joinpoint Regression program (version 4.5.01) from the NCI was utilized to examine annual percent change (APC) in cHCC-CC incidence and IB mortality. APC is one approach to describe trends in incidence or mortality over time. In this way, the cancer incidence or mortality are supposed to change at a constant percentage from the previous year. For cHCC-CC incidence or IB mortality, the curve was fitted using joined log-linear segments; thus, the change of APC in each segment can be obtained.

We used STATA/SE (version 11.0) to perform survival analyses. Kaplan-Meier method was utilized to obtain cumulative survival rates, and log-rank test was utilized to compare survival curves [23, 24].We used the Cox proportional hazards models to perform the independent significance of factors for mortality. The related factors in the study included race, SEER stage, year of diagnosis, treatment, and tumor size. Statistical significance was set at *P* < 0.05.

## Results

### Patient and primary tumor characteristics

Between 2000 and 2014, 645 patients in the SEER-18 database were diagnosed with cHCC-CC, of whom, 643 were eligible for this descriptive analysis (Fig. 1). The mean age of the overall population was 62 years (interquartile range 56–72 years). cHCC-CC was more common in males (*n* = 433; 67.4%) than in females (*n* = 209; 32.6%). Additionally, most patients were Caucasian (*n* = 473; 73.7%), with a small proportion of patients of African-American and other races. Overall, the vast majority of patients are in localized disease (*n* = 256; 39.9%), classified by the SEER staging system. The proportion of patients with localized stage markedly increased (from *n* = 49; 32.3% to *n* = 109; 40.7%) from 2000 to 2014. Furthermore, a considerable proportion of the tumors was of unknown grade (*n* = 286; 44.5%). However, the proportion of the tumors of unknown grade decreased during the study period (from *n* = 73; 48.1% to *n* = 102; 38%). The mean tumor size was 6.3 cm (*n* = 398; 398 valid data points were used to calculate this result). The patient characteristics are reported in Table 1.

**Table 1:** Trends in baseline demographic and pathological characteristics of the study population (2000–2014)

Variable	Total	2000–2004	2005–2009	2010–2014
No. of patients (*n*)*	642	152	222	268
Median age (years)*	62	63	61	63
Gender, *n* (%)*				
Women	209(32.6)	59(38.9)	67(30.2)	83(31.0)
Men	433(67.4)	93(61.1)	155(69.8)	185(69.0)
Race, *n* (%)*				
White	473(73.7)	108(71.1)	163(73.4)	202(75.4)
Black	67(10.4)	18(11.8)	15(6.8)	34(12.7)
Other^▲^	102(15.9)	26(17.1)	44(19.8)	32(11.9)
SEER historic stage, *n* (%)*				
Localized	256(39.9)	49(32.3)	98(44.1)	109(40.7)
Regional	162(25.2)	42(27.6)	54(24.3)	66(24.6)
Distant	164(25.5)	37(24.3)	51(22.9)	76(28.4)
Unstaged^★^	60(9.4)	24(15.8)	19(8.7)	17(6.3)
Grade, *n* (%)*				
Well differentiated	23(3.6)	6(3.9)	9(4.1)	8(3.0)
Moderately differentiated	119(18.5)	22(14.5)	38(17.1)	59(22.0)
Poorly differentiated	193(30.1)	44(28.9)	58(26.1)	91(34.0)
Undifferentiated	21(3.3)	7(4.6)	6(2.7)	8(3.0)
Unknown	286(44.5)	73(48.1)	111(50.0)	102(38.0)

*The units are in parentheses

^▲^Three patients’ are unknown

^★^Fifty-two patient’ stage are unknown

### Overall incidence and mortality trends

**Fig. 2:**
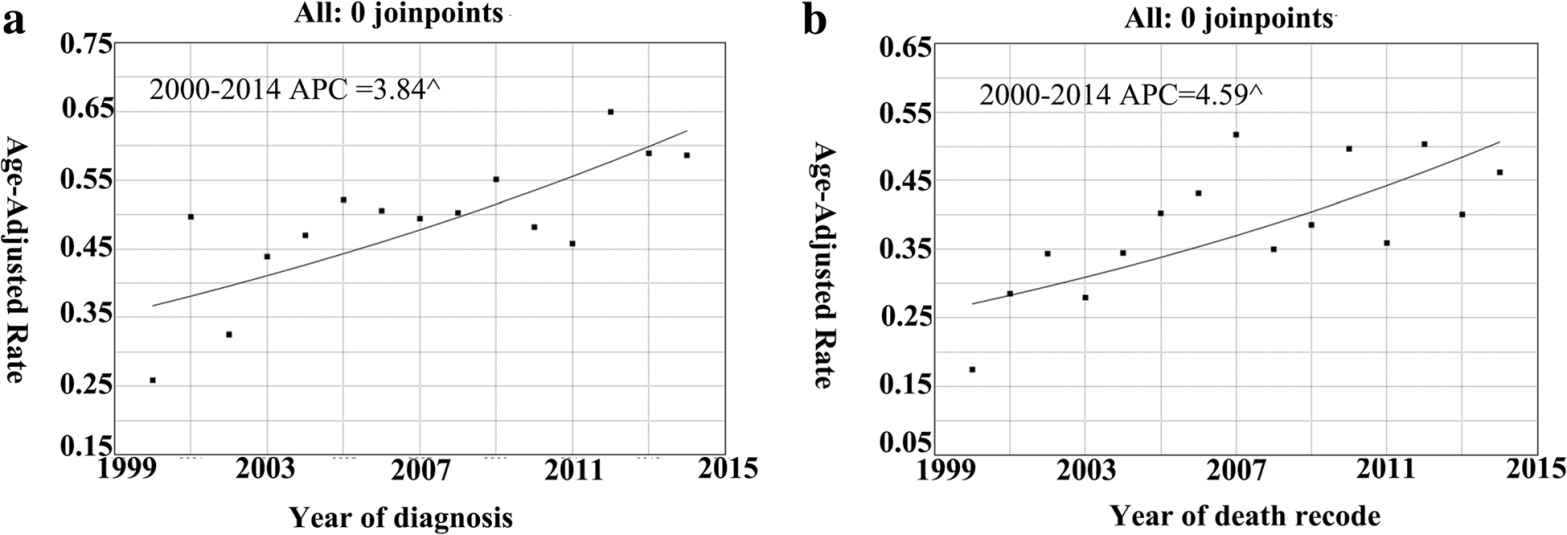
**a** cHCC-CC incidence trends overall 2000–2014. **b** cHCC-CC IB mortality trends overall 2000–2014. ^ mean that *P* < 0.05

The incidence of cHCC-CC displayed an overall increase. The incidence of cHCC-CC was 0.26 per 1,000,000 individuals in 2000 and 0.59 per 1,000,000 individuals in 2014 (Fig. 2a), with an APC of 3.84% (95% confidence interval [CI] 1.7–6.1; *P* < 0.05). Similarly, the IB mortality for cHCC-CC displayed an overall increase. The IB mortality for cHCC-CC was 0.17 per 1,000,000 individuals in 2000 and 0.46 per 1,000,000 individuals in 2014 (Fig. 2b), with an APC of 4.59% (95% CI 1.9–7.4; *P* < 0.05).

### Trends by sex

**Fig. 3:**
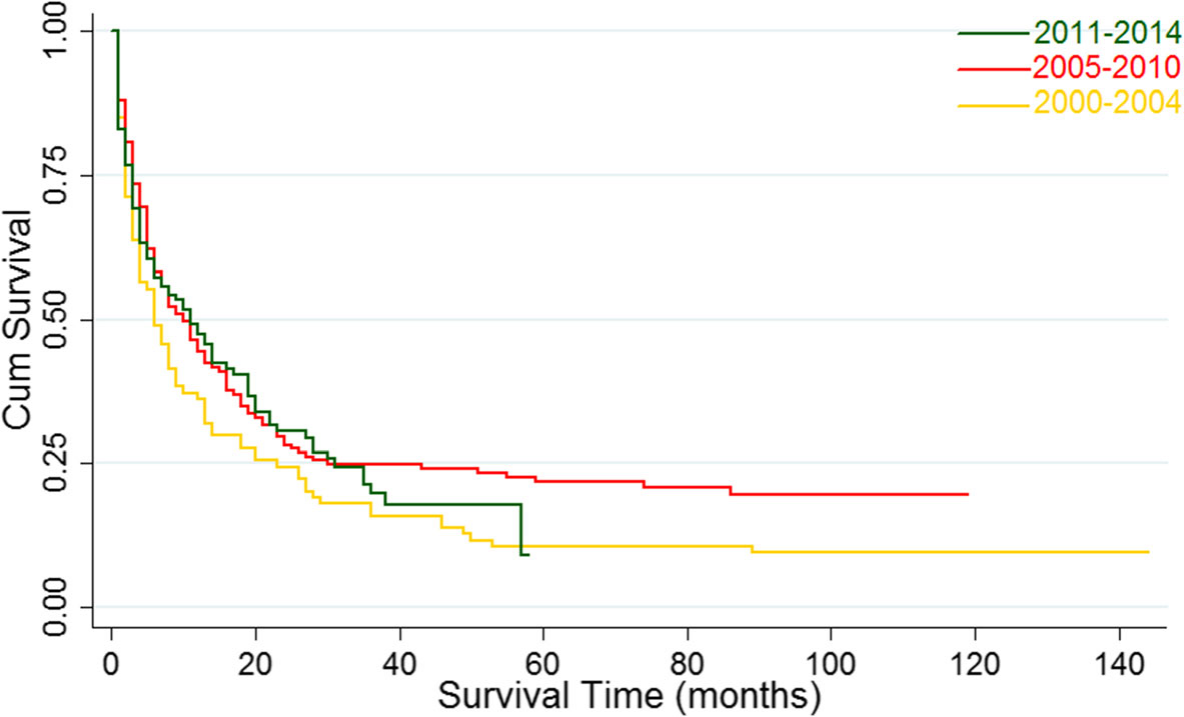
**a** cHCC-CC incidence trends 2000–2014 for men and women, respectively. **b** cHCC-CC IB mortality trends 2000–2014 for men and women, respectively. **c** cHCC-CC incidence trends 2000–2014 for all stage, respectively. **d** cHCC-CC IB mortality trends 2000–2014 for all stage, respectively. ^ mean that *P* < 0.05

For further investigation, we divided the study population into two subgroups according to sex. The incidence of cHCC-CC in males displayed a sustained increase in the whole (Fig. 3a), and it was 0.22 per 1,000,000 individuals in 2000 and 0.88 per 1,000,000 individuals in 2014. During this period, the APC was 5.73% (95% CI 1.6–10.0; *P* < 0.05). As for females, the incidence was 0.27 per 1,000,000 individuals in 2000 and 0.34 per 1,000,000 individuals in 2014. Thus, the APC was lower in females than in males. In addition, the incidence of cHCC-CC in males was slightly higher than that in females. Furthermore, the IB mortality for cHCC-CC followed a similar trend in both males and females (Fig. 3b), with an APC of 7.81% (95% CI 2.1–13.9; *P* < 0.05) in males and a lower APC in females.

### Trends by stage

We further examined the data according to the SEER stage. In general, the incidence of localized cHCC-CC was the highest among the four categories (Fig. 3c), and the incidence of regionalized and distant cHCC-CC was basically similar. The APC of localized cHCC-CC was 5.33% (95% CI 0.7–10.1; *P* < 0.05) from 2000 to 2014, which was considerably high. However, the APC for regionalized and distant cHCC-CC was lower than that for localized cHCC-CC at 4.38% (95% CI 0.6–8.3; *P* < 0.05) and 4.92% (95% CI 1.1–8.8; *P* < 0.05), respectively. In contrast, the incidence of cHCC-CC of the unknown stage displayed an overall decrease, with an APC of − 7.75% (95% CI − 7.8–13.9; *P* < 0.05). Furthermore, the IB mortality (Fig. 3d) for localized and distant cHCC-CC was generally similar and was higher than that for regionalized cHCC-CC. Based on the overall trend, the IB mortality for regional cHCC-CC displayed an APC of 7.71% (95% CI 2.4–13.2; *P* < 0.05) from 2000 to 2014, whereas that for localized and distant, cHCC-CC displayed a lower APC of 5.98% (95% CI 0.3–12.0; *P* < 0.05) and 5.19% (95% CI 0.4–10.2; *P* < 0.05), respectively.

### Long-term survival outcomes

**Fig. 4:**
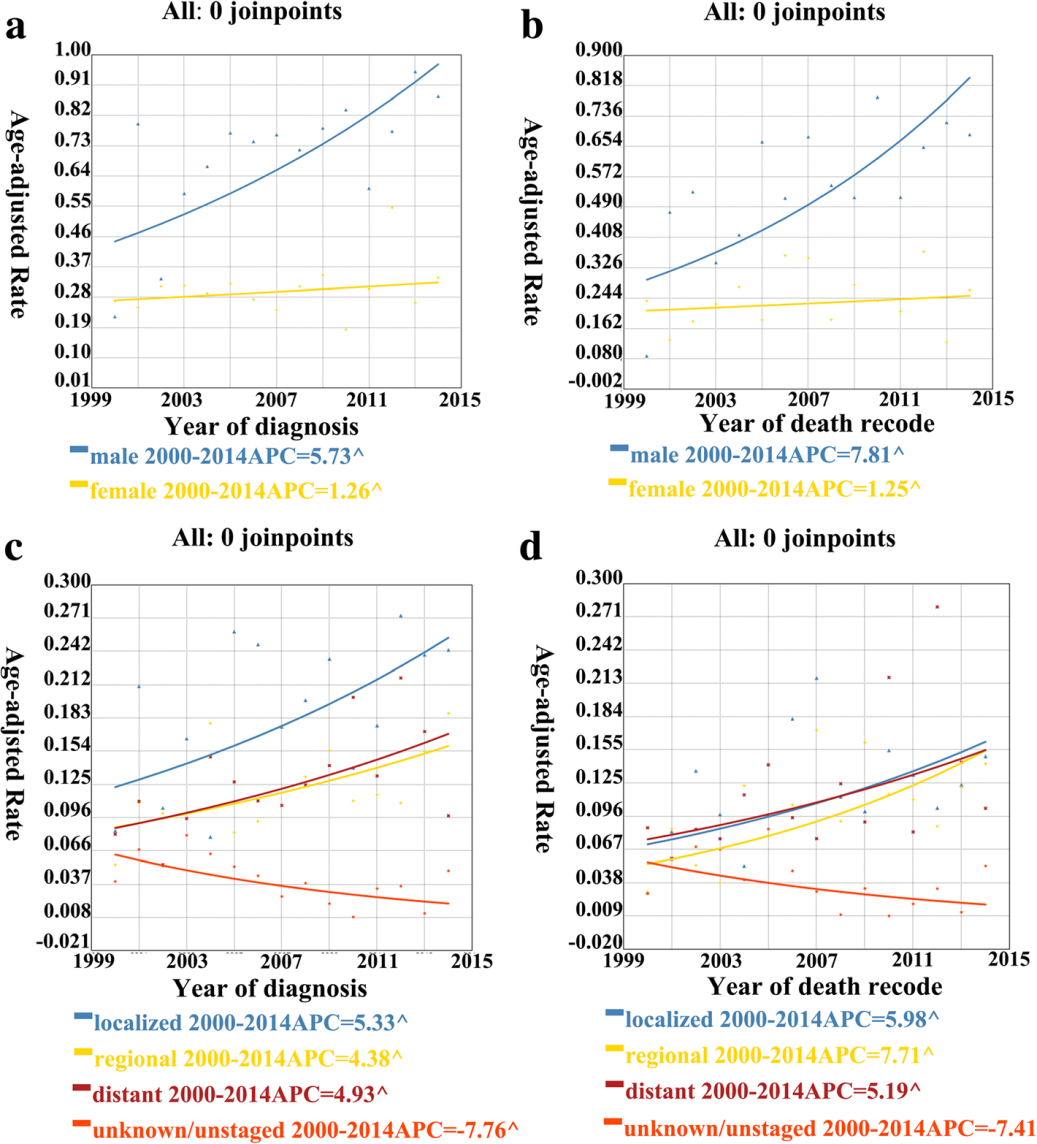
Kaplan-Meier’s analysis: cHCC-CC patients from 2000 to 2014.Graph shows no significant increasing survival from the 2000–2004 to 2010–2014. The *P* values = 0.171

**Fig. 5:**
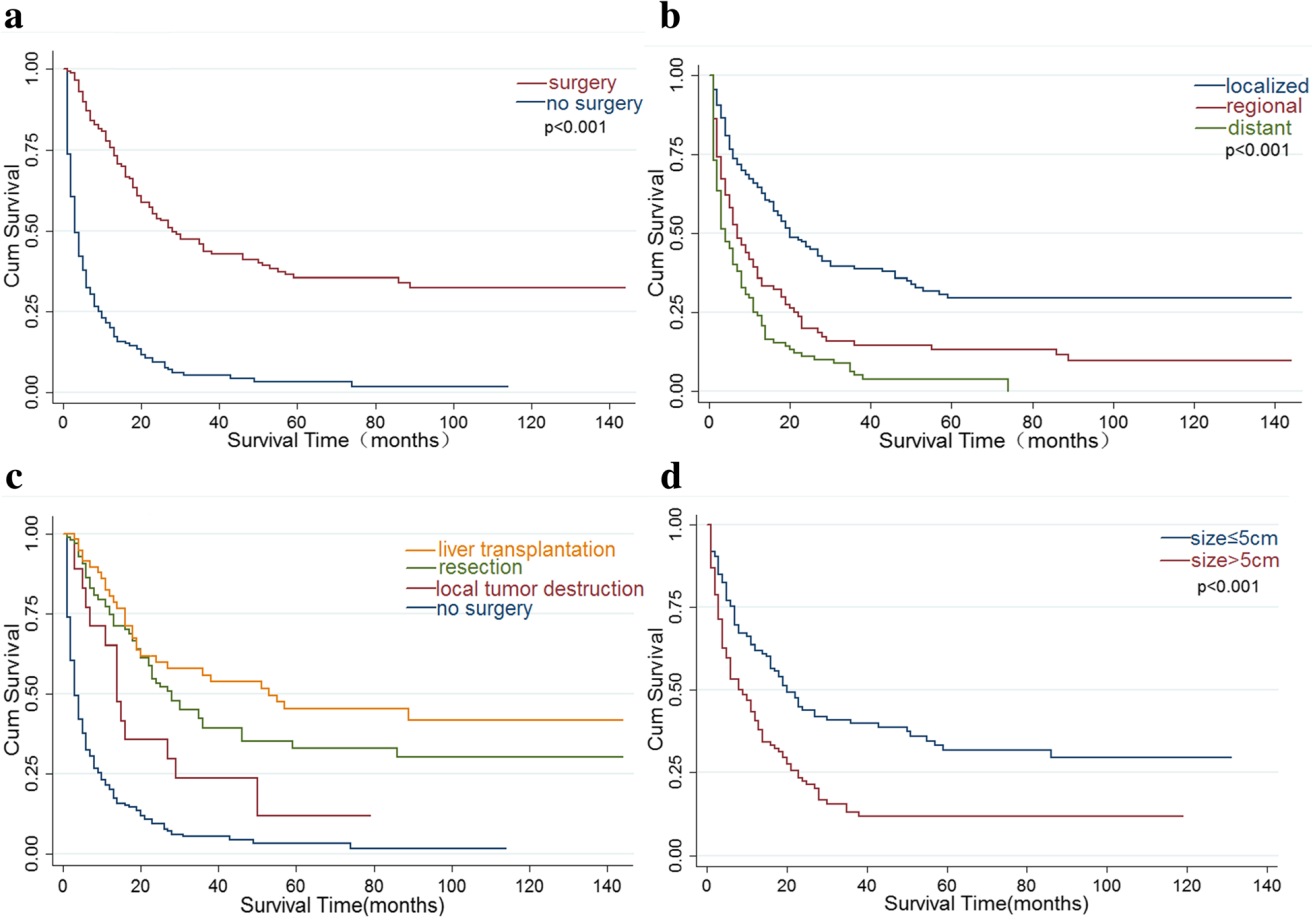
Kaplan-Meier’s analysis. **a** Treatment of cHCC-CC. **b** Localized regional and distant cHCC-CC. Graph shows increasing survival from localized to distant. The *P* values reported for trend analysis refers to comparison among all stage. **c** Specific treatment of cHCC-CC. **d** Tumor size of cHCC-CC

For the total study population, the median survival was 9 months (95% CI 7–11). The 1-, 3-, and 5-year survival rates were 43.4%, 21.5%, and 17.1%, respectively. Although the median survival from 2000 to 2014 increased from 6 months to 11 months, the change was not significant based on the results of the log-rank test (Pp = 0.171) (Fig. 4). The improvement in the median survival was reflected in patients undergoing surgical treatment (3 vs. 28 months; *P* < 0.001) (Fig. 5a). Moreover, a statistically significant increase in median survival was reflected in patients stratified by the SEER stage (the median survival of patients with distant, regionalized, and localized cHCC-HCC was 4 months (95% CI 3–6), 7 months (95% CI: 5–11), and 20 months (95% CI 16–28) respectively, *P* < 0.001) (Fig. 5b) and in patients undergoing LT relative to patients who underwent local tumor destruction (14 vs. 53 months; *P* = 0.014) (Fig. 5c), whereas that of patients with resection was 28 months (95% CI 22–36). However, a decrease in the median survival was observed in patients with tumor size > 5 cm (20 vs. 9 months; *P* < 0.001) (Fig. 5d).

In univariate analysis, African-American race, distant disease, regionalized disease, tumor size ≥ 5 cm, and no surgery were independently associated with mortality. In contrast, localized disease, tumor size < 5 cm, and surgical treatment were associated with positive prognosis. All results are reported in Table 2.

**Table 2:** Univariate Cox’s proportional hazards model assessing factors associated with mortality after diagnosis of cHCC-CC

Risk factor	HR*	995% CI		*P* value
		Lower	Upper	
Race				
Other	Referent			
White	1.19	0.88	1.62	0.26(NS)
Black	1.65	1.07	2.54	0.023
SEER stage				
Localized	Referent			
Regional	1.97	1.47	2.63	< 0.001
Distant	3.09	2.31	4.23	< 0.001
Tumor size, cm				
< 5	Referent			
≥ 5	1.90	1.41	2.56	< 0.001
Treatment				
No surgery	Referent			
Surgery	0.22	0.17	0.29	< 0.001
Year of diagnosis				
2000–2004	Referent			
2005–2009	0.74	0.57	0.99	0.043
2010–2014	0.83	0.62	1.10	0.191(NS)

*HRs greater than 1.0 indicate a higher risk of death

## Discussion

cHCC-CC is a rare malignancy from the liver with distinctive clinicopathological and prognostic features from those of HCC and ICC. The reported frequency of cHCC-CC among cases with primary liver carcinoma varies widely from 0.4 to 14.2% [1, 2, 11, 25–28]. Our study aimed to explore the trends in cHCC-CC incidence, IB mortality, and survival. We also examined the independent predictors of mortality.

Based on our analysis, cHCC-CC incidence continued to increase in the US population during our study period, and the rate of increase in incidence was steady on the whole, at approximately 4.59% per year. The steady increase in the incidence of cHCC-CC might manifest that main prevention strategies for cHCC-CC have not improved much in recent years. The current analysis on the APC of cHCC-CC incidence provides an important support for projecting and assessing cancer control programs. In addition, IB mortality for cHCC-CC displays similar changes. The steady increase in IB mortality may imply that the treatment of cHCC-CC has not improved or progressed much in recent years. More attention should be thus paid to this malignant tumor type, and further studies are warranted to explore therapies to benefit these patients.

In our analysis, males (67.4%) were more commonly affected with cHCC-CC than females, and the patients were apt to be older than 60 years when they were diagnosed (mean age at diagnosis = 62.9 years). In previous studies, cHCC-CC was indicated to be analogous to HCC with respect to demographic and clinical characteristics, with an apparent male dominance and an average age at presentation between 50 and 60 years [17, 29–31]. cHCC-CC had a medium prognosis with respect to HCC and ICC, accord with the results of previous researches [31–34]. In our analysis, the 1-, 3-, and 5-year survival rates were 43.4%, 21.5%, and 17.1%, respectively. Based on the literature, the survival rates for HCC are 49%, 19%, and < 10% at 1, 3, and 5 years, respectively [35], and those for ICC are 38.9%,18.5%, and 14.6% at 1, 3, and 5 years, respectively [36, 37].

Several studies on cHCC-CC have been centered on Asian populations because of the higher incidence of primary liver cancer in Asian [18, 29, 30]. In our study, African-American race was a negative prognostic factor relative to other races (American-Indian/Alaska Native, Asian/Pacific Islander). The results could be explained by differences in risk factors or prevention awareness between these racial groups. Therefore, when screening for liver malignancy in African-American populations, we should be cautious of cHCC-CC rather than being confined to HCC and ICC. These measures may also lead to a shift in health care resources associated with cHCC-CC. The prevention and control measures of HCC may also benefit to cHCC-CC patients. To the best of our knowledge, Asian populations were at a high risk of HCC; thus, national public health department might implement more rigorous and elaborate prevention strategies in these populations. Hence, the prevention and control measures of cHCC-CC may be extrapolated from those of HCC to determine strategies to prevent and control cHCC-CC in general.

In our cohort, surgical treatment (LT and resection) at an early stage generally resulted in a better prognosis than interventions at an advanced stage. The best mean survival was observed among patients undergoing LT for localized disease (86.7 months; 95% CI 66.6–106.9). In addition, patients undergoing resection for localized cHCC-CC had favorable mean survival (68.1 months; 95% CI 49.9–86.3). However, as many patients were already at an advanced stage at diagnosis, they were not candidates for LT or resection. Further investigations and efforts should thus be focused on early diagnosis and treatment. In addition, comprehensive treatment of advanced disease is worth exploring.

Based on our results, tumor size > 5 cm, and regionalized and distant SEER stages were independently associated with an increased risk of death, consistent with the results of previous studies [31–34]. Therefore, these factors indicative of unfavorable prognosis might provide support for the decision of clinical treatment program and risk assessment.

Using data from the SEER database between 1988 and 2009, Mattia Garancini found that in comparison with HCC and ICC, cHCC-CC displays medium demographic, clinical, and survival characteristics, and factors affecting the prognosis of cHCC-CC patients are usually limited to sources of apparent survival benefit [32]. We carried out a more comprehensive assessment of cHCC-CC, such as the analysis of incidence and IB mortality trends by APC and taking advantage of sex and stage to examine trends. However, there are many limitations to this study. Many of the limitations of population-based datasets, such as selection bias and missing information, have been previously described [38]. Furthermore, the SEER database does not provide information on surgical volume and patient comorbidities.

## Conclusions

The diagnosis and management of cHCC-CC are changing in the US population, with adverse mortality and survival outcomes. We identified a steady increase in the overall incidence of cHCC-CC, which indicates that main prevention strategies for cHCC-CC have not improved much in recent years and that cHCC-CC needs to be taken seriously. Our data may thus be helpful for designing and evaluating cancer control programs. The IB mortality data suggest that the treatment of cHCC-CC has not progressed much in recent years, and further studies are warranted to explore therapies to benefit these patients. Additionally, surgical treatment (LT and resection) at an early stage generally results in good prognosis, suggesting that further investigations and efforts should be focused on early diagnosis and treatment and that comprehensive treatment of advanced diseases is worth exploring.

## Abbreviations

APC: Annual percent change
cHCC-CC: Combined hepatocellular-cholangiocarcinoma
HCC: Hepatocellular carcinoma
IB mortality: Incidence-based mortality
ICC: Intrahepatic cholangiocarcinoma
LT: Liver transplantation
RAF: Radiofrequency ablation
SEER: Surveillance, Epidemiology, and End Results
TACE: Transarterial chemoembolization
